# Regulatory single nucleotide polymorphisms (rSNPs) at the promoters 1A and 1B of the human APC gene

**DOI:** 10.1186/s12863-016-0460-8

**Published:** 2016-12-22

**Authors:** Marina Yu Matveeva, Elena V. Kashina, Vasily V. Reshetnikov, Leonid O. Bryzgalov, Elena V. Antontseva, Natalia P. Bondar, Tatiana I. Merkulova

**Affiliations:** 1 Institute of Cytology and Genetics, Siberian Branch, Russian Academy of Sciences, Lavrentieva avenue 10, Novosibirsk, 630090 Russian Federation; 2 Novosibirsk State University, Pirogova street 2, Novosibirsk, 630090 Russian Federation

**Keywords:** Regulatory SNPs, APC, Promoters, EMSA, Luciferase reporter, Oncopathology

## Abstract

**Background:**

Germline mutations in the coding sequence of the tumour suppressor APC gene give rise to familial adenomatous polyposis (which leads to colorectal cancer) and are associated with many other oncopathologies. The loss of APC function because of deletion of putative promoter 1A or 1B also results in the development of colorectal cancer. Since the regions of promoters 1A and 1B contain many single nucleotide polymorphisms (SNPs), the aim of this study was to perform functional analysis of some of these SNPs by means of an electrophoretic mobility shift assay (EMSA) and a luciferase reporter assay.

**Results:**

First, it was shown that both putative promoters of APC (1A and 1B) drive transcription in an in vitro reporter experiment. From eleven randomly selected SNPs of promoter 1A and four SNPs of promoter 1B, nine and two respectively showed differential patterns of binding of nuclear proteins to oligonucleotide probes corresponding to alternative alleles. The luciferase reporter assay showed that among the six SNPs tested, the rs75612255 C allele and rs113017087 C allele in promoter 1A as well as the rs138386816 T allele and rs115658307 T allele in promoter 1B significantly increased luciferase activity in the human erythromyeloblastoid leukaemia cell line K562. In human colorectal cancer HCT-116 cells, none of the substitutions under study had any effect, with the exception of minor allele G of rs79896135 in promoter 1B. This allele significantly decreased the luciferase reporter’s activity

**Conclusion:**

Our results indicate that many SNPs in APC promoters 1A and 1B are functionally relevant and that allele G of rs79896135 may be associated with the predisposition to colorectal cancer.

**Electronic supplementary material:**

The online version of this article (doi:10.1186/s12863-016-0460-8) contains supplementary material, which is available to authorized users.

## Background

The adenomatous polyposis coli (APC) gene is mapped to chromosome 5q and encodes a protein consisting of 2843 amino acid residues that has been implicated in various cellular functions [1, 2]. In particular, the APC protein is best known as a negative regulator of the transcription factor (TF) β-catenin, an effector of the Wnt signaling pathway [3]. As a component of this signaling pathway, APC participates in a multiprotein ‘destruction complex’ that targets the proto-oncogene β-catenin for ubiquitin-mediated proteolysis [4]. The loss of APC function leads to translocation of β-catenin from the lateral cell membrane to the nucleus, where it promotes transcription of multiple genes involved in tumour growth and invasion [2].

Germline mutations in this tumour suppressor gene (APC) give rise to familial adenomatous polyposis (FAP). The latter is an autosomal-dominant colorectal-cancer predisposition syndrome and accounts for ~1% of newly diagnosed cases of colorectal cancer. FAP is characterised by the development of multiple adenomatous polyps (from hundreds to thousands) in the large intestine [5]. The great majority of the mutations observed in the APC gene in patients with FAP are detected in the coding part of the gene. Usually, these are frameshift mutations caused by insertions or deletions of nucleotides and point mutations that predominantly generate a truncated APC peptide. Most of known mutations in APC affect codons 1250–1464 [6, 7].

Many other oncopathologies are also associated with mutations in APC’s coding sequence. For example, in the case of pancreatic acinar cell carcinomas, there is a report of a frameshift mutation characterised by an insertion of 170 nucleotides after nucleotide 4177 and resulting in a truncated protein [8]. Likewise, the molecular basis of Turcot syndrome (colonic polyposis and a primary brain tumour in the same patient) is truncated variants of the APC protein [9]. Moreover, mutations in APC’s coding regions were detected in 23% of cases of ileal enterochromaffin cell neuroendocrine neoplasms; in particular, 57% were missense and 14% were nonsense or frameshift mutations [10]. Mutations in exon 15 of the APC gene were detected in 22.1% of cases of gastric cancer [11].

In addition to mutations in the coding sequence, a loss of APC gene function can occur through alternative genetic and epigenetic mechanisms such as promoter deletion or methylation. At present, there are two known alternative start sites for APC that are located 17 and 47 kb upstream of the initiating methionine codon, respectively; from these start sites, alternative mRNAs containing exon 1A or 1B are transcribed. By default it is believed that these transcripts originate in alternative promoters 1A and 1B [12] although direct evidence for the existence of these promoters has not been obtained yet. It is known that big deletions encompassing any of these start sites cause FAP. Thus, Charames et al. identified a germline deletion corresponding to promoter 1A and 5′ untranslated regions of *APC* in 28-year-old proband of Canadian Mennonite FAP family. This large deletion results in allele silencing and was also detected among the proband’s other clinically affected siblings while the unaffected ones do not carry the same deletion [13]. Then a deletion (61 kb) encompassing half of promoter 1B was identified in the largest family of the Swedish Polyposis Registry, that leads to an imbalance in allele-specific APC expression. The deletions were detected in all of the affected 11 individuals, but not in the normal controls (50 individuals) [14]. Additionally a novel ~11 kb deletion that encompasses the APC 1B promoter and exon was detected in affected (but not in unaffected) family members of three kindreds from USA FAP registry. This deletion was accompanied by silencing of one of the APC alleles as well [15] Hypermethylation of putative promoter 1A has also been reported for familial polyposis and human colorectal cancer [16, 17].

According to dbSNP NCBI, the regions of promoters 1A and 1B contain a multitude of single nucleotide polymorphisms (SNPs). It is well known that if SNPs that are located in promoter regions of candidate genes change a TF’s binding pattern or the affinity for these proteins and thus influence the level of gene transcription, then they may be promising biomarkers of genetic predisposition to various diseases [18–20]. Nevertheless, SNPs of APC promoters are still poorly studied. Therefore, in the present study, we conducted a functional analysis of some SNPs located in the regions of promoters 1A and 1B; this analysis included assessment of the influence of nucleotide substitutions on binding patters of nuclear proteins in an electrophoretic mobility shift assay (EMSA) and on activity of these promoters in luciferase reporter experiments.

## Methods

### Cell lines, nuclear extract preparation and electrophoretic mobility shift assays

The cultivation of cell lines (human hepatoma cells HepG2, human cervical adenocarcinoma cells HelaS3, human erythromyeloblastoid leukemia cells K562, human colorectal cancer cells HCT-116), preparation of nuclear extracts and Electrophoretic Mobility Shift Assays (EMSA) has been described previously [21].

### Construction of plasmids containing 1A и 1B APC promoter regions

To construct the target 1A и 1B APC promoter-reporter plasmids, we synthesized the DNA fragments by amplifying the 517-bp 1B promoter region (chr5:112042901–112043417; GRCh37/hg19) and the 435-bp 1A promoter region (chr5:112073194–112073721; GRCh37/hg19) using HCT-116 genomic DNA as a template. The primers were 5′- CTCTCTCGAGTCATCTTTCTATCATCAGCGTCTA −3′ (1BXho forward) and 5′- ACCCAAGCTTATAGGGGGCGCCGAGGCC −3′ (1BHind reverse) CTCTCTCGAGGTGCTGCAAAAATCATAGCAATCG −3′ (APCXhoI forward) and 5′- ACCCAAGCTTTGTGCCAAGGAAAGGCCATC −3′ (APCHindIII reverse) correspondingly. To facilitate plasmid construction, two endonuclease sites, XhoI and HindIII were inserted to both ends of the amplicon (underlined sequences). PCR was performed as follows: 200 μM dNTP; 10-x As-buffer (65 мM Tpиc-HCl pH 8,9; 1,6 мM (NH4)2SO4; 0,05% Tween 20, 15мM MgCl2); 2,5 pmole of reverse APCHind primer + forward APCXho primer or 2.5 pmole of forward 1BXho primer + reverse 1BHind primer; 0,25 μg ДHК; 1 U Taq - polymerase (SibEnzyme, Russia). The amplified fragment was digested with XhoI and HindIII, and then cloned into the luciferase expression vector pGL4.10[luc2] (Promega) with T4 ligase. The constructs were all confirmed by DNA sequencing.

### Site-directed mutagenesis

The 6 pairs of mutagenic oligonucleotide primers used in this study are listed in Table 2, which were designed to amplify the mutant fragments. These primer pairs were overlapped by 2/3 of the length, with mutated bases at the position 8 in the direction of 5'- > 3'. PCR reaction was performed in a two steps. As a first PCR template the promoter-reporter plasmids pGL4.10 - 1A and pGL4.10 – 1B were used. The first PCR mixture contained 200 μM dNTP; 10-x As-buffer (65 мM Tpиc-HCl pH 8,9; 1,6 мM (NH4)2SO4; 0,05% Tween 20, 15мM MgCl2); 2,5 pmole of reverse APCHind or of reverse 1BHind primer + forward mutagenic oligonucleotide primer (step 1–1) or 2.5 pmole of forward APCXho primer or of forward 1BXho primer + reverse mutagenic oligonucleotide primer (step 1–2); 0,25 μg ДHК; 1 U Taq - polymerase (SibEnzyme, Russia). As the second PCR template amplicons from the first PCR reactions were used adds primers. The presence of the mutations was confirmed by Sanger′s sequencing as described [22].

### Luciferase reporter assay

For luciferase reporter assays HCT −116 and K562 cells were used. The plasmids under study were co-transfected with pRL-TK using Screen FectA (Incella GmbH, Germany). Luciferase activity was measured 24 h after transfection by using the dual-luciferase reporter assay kit (Promega, USA).

### Statistical analysis

STATISTICA 6 was used for statistical analysis.

## Results

### Both putative 1A и 1B *APC* promoters drive transcription in the in vitro reporter assay

**Fig. 1:**
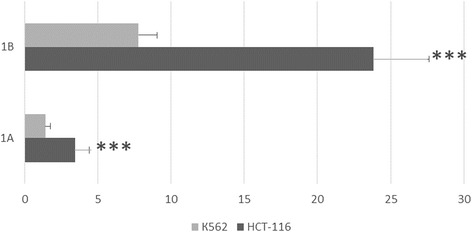
Analysis of activity of APC promoters in the reporter constructs. The luciferase activity was measured by the dual-luciferase reporter assay kit (Promega, USA). Firefly luciferase activity was normalized to Renilla luciferase activity. The bars indicated the mean ± SE of the luciferase activity (*N* = 7)

Previously, we developed a method for whole-genome identification of regulatory regions on the basis of the assumption that enrichment of a genomic region for peaks from high-throughput sequencing of chromatin immunoprecipitation material (ChIP-Seq) indicates that this is a regulatory region [21]. Application of this approach to APC revealed large clusters of ChIP-Seq peaks (up to 28) in two distinct regions (Additional file 1). These regions include start sites for APC’s alternative transcripts containing exon 1A or 1B, which apparently originate in alternative promoter 1A or 1B [12]. To date, there are some data on the expression patterns of the above-mentioned alternative transcripts of APC in various organs and tissues [13, 23], but experiments on direct study of activity of promoters 1A and 1B have not been conducted. To close this gap, we created reporter constructs containing either a distal (1B) (−301; +216; chr5: 112042901–112043417) or proximal (1A) (−315; +120; chr5: 112073194–112073721) APC promoter by cloning of the corresponding fragments into the promoterless plasmid pGL4.10. The results showed that after transfection of the K562 cells with one of these plasmid constructs, expression of the luciferase reporter gene under control of promoter 1A was fourfold stronger than that of the reference promoterless plasmid pGL4.10. After transfection with the plasmid containing promoter 1B, the relative luciferase activity was 22-fold greater than the reference activity (Fig. 1). Therefore, activity of promoter 1B was approximately sixfold higher than that of promoter 1A.

Similar results were obtained with HCT-116 cells although in this case, both promoters showed even higher activity. In particular, under the control of promoter 1A, expression of the luciferase gene increased 10-fold, whereas under control of promoter 1B as much as 80-fold in comparison with the reference plasmid (pGL4.10; Fig. 1). The two constructs differed by the factor of 8, which approximately matches the difference in activity of promoters 1A and 1B in K562 cells.

Overall, this analysis revealed that, when cloned into a promoterless plasmid, the above-mentioned regions of the APC gene indeed behave as a promoters in transfection experiments, and promoter 1B was found to enhance expression of the reporter gene more strongly than promoter 1A did.

### Influence of SNPs from promoters 1A and 1B on nuclear proteins binding

**Fig. 2:**
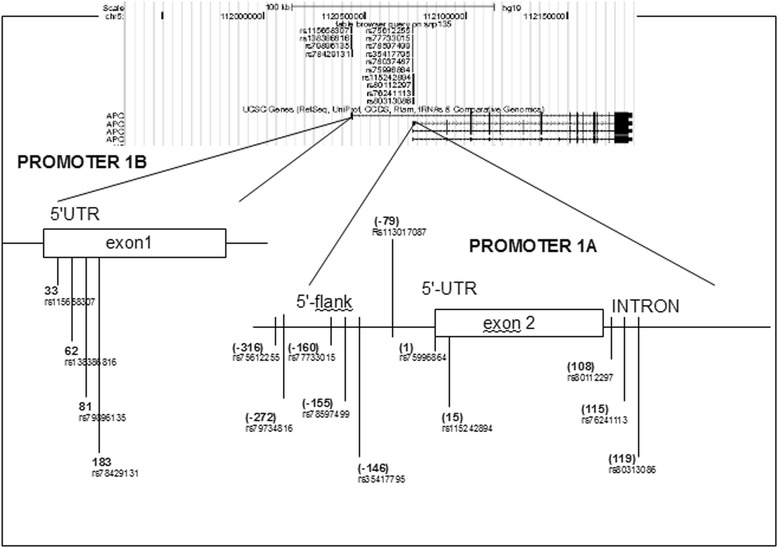
The scheme of location of the two APC promoter regions (*1A* and *1B*) and the position of the SNPs selected in the present study

According to data from dbSNP NCBI [24] there are many SNPs in promoter regions of APC. For empirical analysis, we randomly selected 15 SNPs (rs75612255, rs79734816, rs77733015, rs78597499, rs35417795, rs113017087, rs75996864, rs115242894, rs80112297, rs76241113, rs80313086, rs115658307, rs138386816, rs79896135, and rs78429131) (Fig. 2). For each of them, double-stranded oligonucleotides (corresponding to alternative alleles) were synthesised (Table 1). These oligonucleotides were then used as DNA probes in EMSA experiments with nuclear extracts from four human cell lines (HepG2, HeLaS3, HCT-116, and K562). We used two to three independent replicates in each EMSA.

**Table 1:** List of oligonucleotide probes tested in EMSA

SNP identifier in dbSNP NCBI database	Sequences of oligonucleotides 5’- > 3’	Coordinates (hg19)	Minor Allele Count (MAF)	Promoter
Rs75612255: T > C	Allele T: ATTTATTACTCTCCC*T*CCCACCTCCGGCATC Allele C: ATTTATTACTCTCCC*C*CCCACCTCCGGCATC	Chr5:112737543 (−316), 5'-flank	NA	1A
Rs79734816: T > C	Allele T: TCTGCCCTGCGGACC*T*CCCCCGACTCTTTAC Allele C: TCTGCCCTGCGGACC*C*CCCCCGACTCTTTAC	Chr5: 112737587 (−272), 5'-flank	NA	1A
Rs77733015: T > G	Allele T: GGGCTAGGCAGGCTG*T*GCGGTTGGGCGGGGC Allele G: GGGCTAGGCAGGCTG*G*GCGGTTGGGCGGGGC	Chr5: 112737699 (−160), 5'-flank	NA	1A
Rs78597499: T > G	Allele T: AGGCAGGCTGTGCGG*T*TGGGCGGGGCCCTGT Allele G: AGGCAGGCTGTGCGG*G*TGGGCGGGGCCCTGT	Chr5: 112737704 (−155), 5'-flank	NA	1A
Rs35417795: 112737713delG	Allele del: GTGCGGTTGGGCGGG*-*CCCTGTGCCCCACTG Allele G: GTGCGGTTGGGCGGG*G*CCCTGTGCCCCACTG	Chr5: 112737713 (−146), 5'-flank	NA	1A
Rs113017087: T > C	Allele T: GTGTAATCCGCTGGA*T*GCGGACCAGGGCGCT Allele C: GTGTAATCCGCTGGA*C*GCGGACCAGGGCGCT	Chr5: 112737780 (−79), 5'-flank	C = 0.0032/16	1A
Rs75996864: T > G	Allele C: ACCGACATGTGGCTG*T*ATTGGTGCAGCCCGC Allele G: ACCGACATGTGGCTG*G*ATTGGTGCAGCCCGC	Chr5: 112737860 (1), 5'-UTR	NA	1A
Rs115242894: G > C	Allele G: GTATTGGTGCAGCCC*G*CCAGGGTGTCACTGG Allele C: GTATTGGTGCAGCCC*C*CCAGGGTGTCACTGG	Chr5: 112737874 (15), 5'-UTR	C = 0.0042/21	1A
Rs80112297: A > G	Allele A: GCTTGCTGCGGGGGG*A*GGGGGGAAGGTGGTT Allele G: GCTTGCTGCGGGGGG*G*GGGGGGAAGGTGGTT	Chr5: 112737967 (108), intron	NA	1A
Rs76241113: A > G	Allele A: GCGGGGGGAGGGGGG*A*AGGTGGTTTTCCCTC Allele G: GCGGGGGGAGGGGGG*G*AGGTGGTTTTCCCTC	Chr5: 112737974 (115), intron	NA	1A
Rs80313086: T > G	Allele T: GGGGAGGGGGGAAGG*T*GGTTTTCCCTCGCAC Allele G: GGGGAGGGGGGAAGG*G*GGTTTTCCCTCGCAC	Chr5: 112737978 (119), intron	NA	1A
Rs115658307: C > T	Allele C: CAAGATGGCGGAGGG*C*AAGTAGCAAGGGGGC Allele T: CAAGATGGCGGAGGG*T*AAGTAGCAAGGGGGC	Chr5: 112707537 (33), 5'-UTR	T = 0.0054/27	1B
Rs138386816: C > T	Allele C: GCGGGGTGTGGCCGC*C*GGAAGCCTAGCCGCT Allele T: GCGGGGTGTGGCCGC*T*GGAAGCCTAGCCGCT	Chr5: 112707566 (62), 5'-UTR	T = 0.0088/44	1B
Rs79896135: C > G	Allele C: AGCCTAGCCGCTGCT*C*GGGGGGGACCTGCGG Allele G: AGCCTAGCCGCTGCT*G*GGGGGGGACCTGCGG	Chr5: 112707585 (81), 5'-UTR	G = 0.1777/890	1B
Rs78429131: T > G	Allele T: AGGAAGGTGAAGCAC*T*CAGTTGCCTTCTCGG Allele G: AGGAAGGTGAAGCAC*G*CAGTTGCCTTCTCGG	Chr5: 112707687 (183), 5'-UTR	G = 0.0767/384	1B

**Fig. 3:**
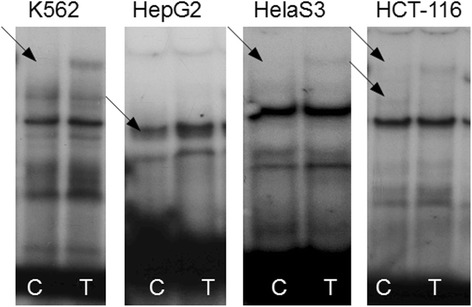
Rs75612255: T > C alters the binding patterns of nuclear proteins from HeLaS3, HepG2, HCT-116, and K562 cells. Changes in the binding of allelic variants with the nuclear proteins are indicated by arrows

The typical example of changes in a binding pattern is shown in Fig. 3. One can see that when nuclear extracts from HeLaS3, HCT-116, and K562 cells were used, allele C (rs75612255) differed from allele T by the absence of the highest retarded band. When HepG2 cell extract was used, this band was not observed for both alleles, apparently as a result of the absence of the corresponding TF in these cells. On the other hand, in the binding assay of proteins from this cell line, a new retarded band appeared, and its intensity was much lower in the case of allele C.

Final results of EMSAs are shown in Table 1. A result was deemed positive (labelled with the + sign) if at least one cell line showed a change in the binding pattern of nuclear proteins. Such changes were observed for 11 (rs75612255, rs79734816, rs35417795, rs113017087, rs7599864, rs115242894, rs80112297, rs76241113, rs80313086, rs138386816, and rs79896135) of the 15 SNPs tested. This finding suggests that the SNPs in question can destroy (or create) a binding site for some TFs and/or enhance or weaken the binding sites for other TFs, and under appropriate conditions, may influence the regulation of gene expression at the transcriptional level.

For experiments with the luciferase system, we selected two SNPs from each alternative promoter - rs75612255: T > C and rs113017087: T > C (from 1A) and rs138386816: C > T and rs79896135: C > G (from 1B)—that showed differential patterns of binding of nuclear proteins to oligonucleotide probes representing the alternative alleles. Besides, we analysed two SNPs from promoter 1B (rs115658307: C > T and rs78429131: T > G) that did not change the protein binding pattern, because it is known that substitutions that do not affect the binding of nuclear-extract proteins can nevertheless influence the expression of a reporter [22].

### Effect of SNPs from promoters 1A and 1B on transcriptional activity

To evaluate the effects of rs75612255 T > C, rs113017087 T > C, rs138386816: C > T, rs79896135: C > G, rs115658307: C > T, and rs78429131: T > G on transcriptional activity, we prepared a series of reporter plasmids containing the corresponding substitutions in promoter 1A or 1B (Table 2). For transfection experiments, we used the human colorectal cancer HCT-116 cells and the human erythromyeloblastoid leukaemia cell line K562.

**Table 2:** Primers for site-directed mutagenesis

SNP	Mutagenic oligonucleotide primer 5’- > 3’
rs115658307: C > T	Forward:GGAGGG*T*AAGTAGCAAGGGGGCGG Reverse:GCTACTT*A*CCCTCCGCCATCTTGTGGG
rs138386816: C > T	Forward:GGCCGC*T*GGAAGCCTAGCCGCTGCT Reverse:GCTTCC*A*GCGGCCACACCCCGCCC
rs79896135: C > G	Forward:GCTGCT*G*GGGGGGGACCTGCGGGCT Reverse:CCCCCC*C*AGCAGCGGCTAGGCTTCC
rs78429131:T > G	Forward:AAGCAC*G*CAGTTGCCTTCTCGGGC Reverse:CAACTG*C*GTGCTTCACCTTCCTCA
rs75612255: T > C	Forward:CTCTCCC*C*CCCACCTCCGGCAT Reverse:AGGTGGG*G*GGGAGAGTAATAAATTA
rs113017087: T > C	Forward:GCTGGA*C*GCGGACCAGGGCGCTCCCC Reverse:GTCCGC*G*TCCAGCGGATTACACAGC

Note: The points of site-direct mutagenesis is marked in italic

**Fig. 4:**
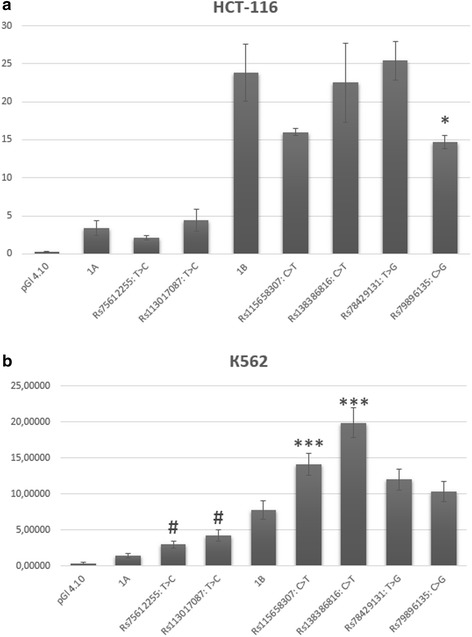
Analysis of activity of reporter constructs. The luciferase activity was measured by the dual-luciferase reporter assay kit (Promega, USA). Firefly luciferase activity was normalized to Renilla luciferase activity. The bars indicated the mean ± SE of the luciferase activity (*N* = 3–7). **a** Cell line HCT-116. Significant differences * *p* < 0,05 was assessed by Students *t* test, compared to promoter *1B* (Student *t*-test). **b** Cell line K562. Significant differences *** *p* < 0,001 was assessed by Students *t* test, compared to promoter *1B*. Significant differences # *p* < 0,05 was assessed by Students *t* test, compared to promoter *1A*

According to the data in Fig. 4, the majority of SNPs under study were functionally active in a luciferase reporter assay, however pointing the obvious cell-specific effects. In K562 cells, the rs75612255 C allele and rs113017087 C allele of promoter 1A as well as alleles rs138386816 T and rs115658307 T of promoter 1B significantly increased luciferase activity, whereas in HCT-116 cells, there was no difference in the activity of the promoters containing alternative alleles at these positions. In contrast, minor allele G of rs79896135 from promoter 1B, which has no effect on the reporter gene expression in K562 cells, significantly decreased the luciferase activity in HCT-116 cells. Among these five SNPs, four (rs75612255, rs113017087, rs138386816, and rs79896135) also yielded differential binding patterns of nuclear proteins in EMSAs. Rs115658307 manifested a functional activity only in a luciferase reporter assay. This may be because the transfection experiments were performed on live cells, whereas EMSA was an in vitro (cell-free) method. Preparation of nuclear extracts for an EMSA is time-consuming, and some nuclear proteins may lose activity by the end of this procedure. Accordingly, the reporter assays may be more informative. Rs78429131 was found to have no effect in both assays of functional activity.

## Discussion

*APC* (adenomatous polyposis coli) was originally identified as a gene mutated in colorectal cancers associated with the FAP syndrome, hence the name [1, 25]. Nowadays, the APC protein is considered a universal tumour suppressor mostly acting as an antagonist of the Wnt signaling pathway [26–28]. Moreover, a growing body of evidence supports the idea that APC performs numerous functions outside the Wnt pathway: e.g., roles in cell migration [29], adhesion [30], chromosome segregation [31], apoptosis [32], and neuronal differentiation [33]. Multifunctionality of the APC protein and the complex expression pattern of its gene [13, 23] are suggestive of fairly complicated organisation of the regulatory regions in the APC gene. So far, however, there is almost no information about these regions.

In the present study, we for the first time obtained direct evidence of promoter activity of the distal (1B) (−301; +216; Chr5: 112042901–112043618) and proximal (1A) (−315; +120; chr5: 112073194–112073721) regions of APC, which include start sites for APC’s alternative transcripts containing exon 1A or 1B. For these experiments, we cloned these regions into the luciferase expression vector pGL4.10. The analysis was conducted on two human cell lines: the colorectal cancer HCT-116 cells and erythromyeloblastoid leukaemia K562 cells: in both cases, activity of promoter 1B was substantially (approximately sixfold-eightfold) higher than that of promoter 1A. This result is consistent with the in vivo data showing that the levels of expression from promoter 1B are 15- to 250-fold higher as compared with transcripts generated from promoter 1A in healthy human tissues (gastric and colorectal mucosa, blood, brain, and small intestine) [13, 23]. In our experiments, the differences in the level of transcription driven by promoters 1B and 1A were found to be more modest; this result may have something to do with insufficient size of the regions inserted into the plasmids and/or differences in the set of regulatory proteins between tissues of a live individual and cultured cells. Be that as it may, our results also clearly show greater activity of promoter 1B.

Next, we studied eleven randomly selected SNPs of promoter 1A and four from promoter 1B by the EMSA. Nuclear extracts were prepared from four human cell lines (HepG2, HeLaS3, HCT-116, and K562). These cell lines originate from different tissues; this situation expanded the range of nuclear proteins that can bind to specific DNA sites. The results of the EMSA revealed that respectively nine and two of the selected SNPs—in at least one cell line—manifest substantial differences in patterns of binding of nuclear proteins to oligonucleotide probes corresponding to alternative alleles. These differences represented either an increase or decrease in the affinity of some TFs, or even disappearance or appearance of binding sites for TFs. Such alterations in TF binding patterns can strongly influence gene expression [22, 34–36]. Indeed, the luciferase reporter assay suggested that all four analyzed SNPs (which demonstrated differential binding patterns in the EMSA) were functionally active. The most interesting among them was minor allele G (MAF = 0.18) of rs79896135 from promoter 1B; this allele decreased luciferase activity 1.5-fold in the human colorectal cancer HCT-116 cells. Although APC promoter 1B for a long time had been thought to play a minor role in the regulation of this gene, several large deletions that encompass this promoter were found to cause allelic silencing and are known to cause FAP. Our data indicate the possibility of such a negative effect of the substitution C > G (rs79896135) on APC expression in vivo and development of predisposition to FAP, and subsequently colorectal cancer. There are some other interesting substitutions in promoter 1B (rs138386816: C > T and rs115658307: C > T) and in promoter 1A (rs75612255: T > C and rs113017087: T > C), which, on the contrary, increased the luciferase reporter activity. These findings are suggestive of a possible oncoprotective effect of these substitutions.

## Conclusion

Overall, our functional analysis uncovered new possible mechanisms of resistance/susceptibility to oncopathologies with involvement of APC because we demonstrated the effect of a number of rare SNPs located in the alternative promotes of this gene both on nuclear protein binding and promoter activity.

## Additional file

Additional file 1: The large clusters of ChIP-Seq peaks is in two distinct regions of *APC* gene (promoter 1A and promoter 1B). (PNG 44 kb)
