# Supplementary figures and images for: Regulatory single nucleotide polymorphisms (rSNPs) at the promoters 1A and 1B of the human APC gene
Source: BMC Genet. 2016 Dec 22;17(Suppl 3):154. doi: 10.1186/s12863-016-0460-8 (PMC5249005; doi:10.1186/s12863-016-0460-8)

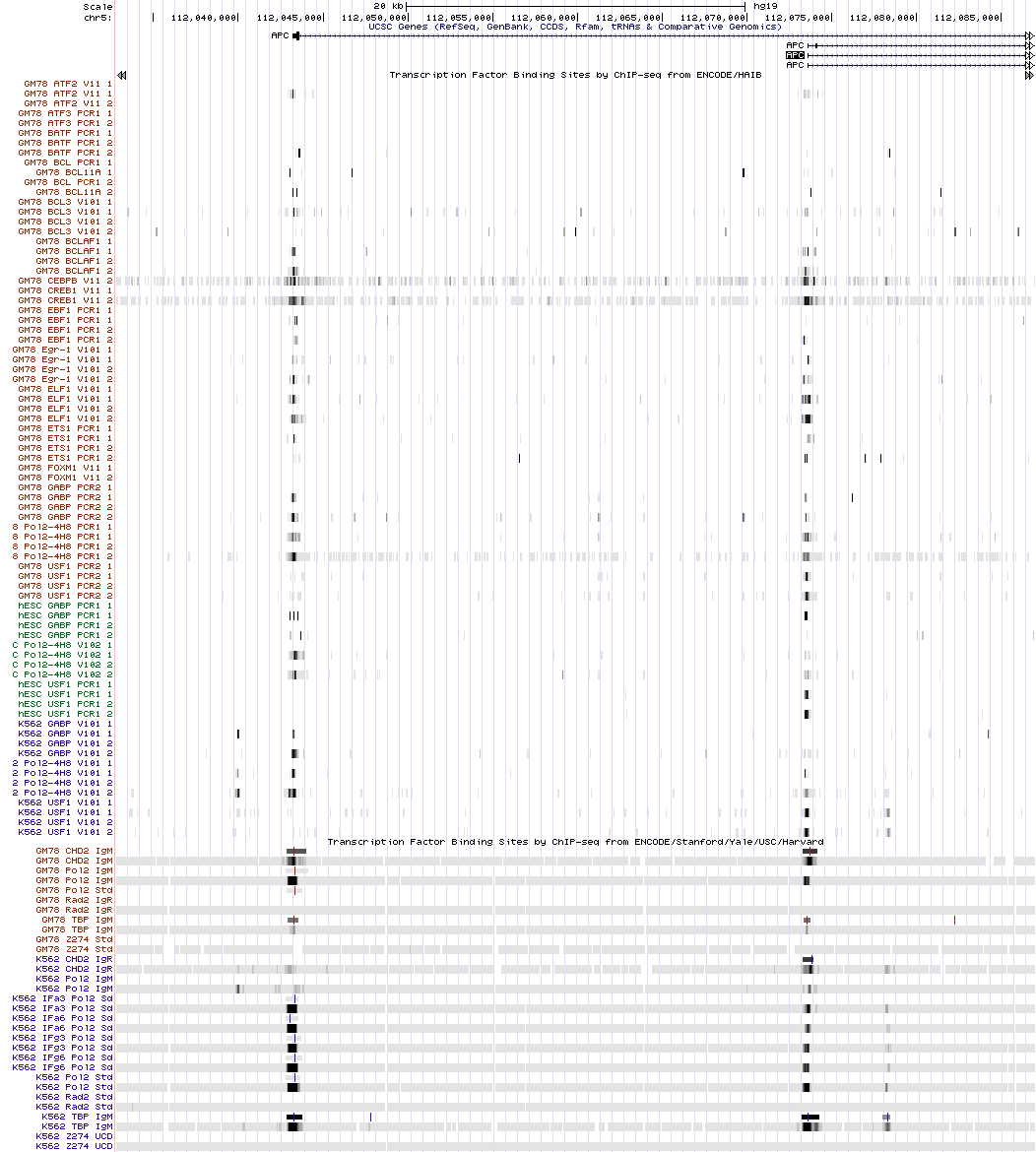

Supplement: Additional file 1: — The large clusters of ChIP-Seq peaks is in two distinct regions of APC gene (promoter 1A and promoter 1B). (PNG 44 kb) [file 12863_2016_460_MOESM1_ESM.png]
